# Validation of radiologists’ findings by computer-aided detection (CAD) software in breast cancer detection with automated 3D breast ultrasound: a concept study in implementation of artificial intelligence software

**DOI:** 10.1177/0284185119858051

**Published:** 2019-07-19

**Authors:** Jan CM van Zelst, Tao Tan, Ritse M Mann, Nico Karssemeijer

**Affiliations:** Department of Radiology and Nuclear Medicine, Radboud University Medical Centre, the Netherlands

**Keywords:** Breast, neoplasm, ultrasound, artificial intelligence, computer-aided detection, screening

## Abstract

**Background:**

Computer-aided detection software for automated breast ultrasound has been shown to have potential in improving the accuracy of radiologists. Alternative ways of implementing computer-aided detection, such as independent validation or preselecting suspicious cases, might also improve radiologists’ accuracy.

**Purpose:**

To investigate the effect of using computer-aided detection software to improve the performance of radiologists by validating findings reported by radiologists during screening with automated breast ultrasound.

**Material and Methods:**

Unilateral automated breast ultrasound exams were performed in 120 women with dense breasts that included 60 randomly selected normal exams, 30 exams with benign lesions, and 30 malignant cases (20 mammography-negative). Eight radiologists were instructed to detect breast cancer and rate lesions using BI-RADS and level-of-suspiciousness scores. Computer-aided detection software was used to check the validity of radiologists' findings. Findings found negative by computer-aided detection were not included in the readers’ performance analysis; however, the nature of these findings were further analyzed. The area under the curve and the partial area under the curve for an interval in the range of 80%–100% specificity before and after validation of computer-aided detection were compared. Sensitivity was computed for all readers at a simulation of 90% specificity.

**Results:**

Partial AUC improved significantly from 0.126 (95% confidence interval [CI] = 0.098–0.153) to 0.142 (95% CI = 0.115–0.169) (*P* = 0.037) after computer-aided detection rejected mostly benign lesions and normal tissue scored BI-RADS 3 or 4. The full areas under the curve (0.823 vs. 0.833, respectively) were not significantly different (*P* = 0.743). Four cancers detected by readers were completely missed by computer-aided detection and four other cancers were detected by both readers and computer-aided detection but falsely rejected due to technical limitations of our implementation of computer-aided detection validation. In this study, validation of computer-aided detection discarded 42.6% of findings that were scored BI-RADS ≥3 by the radiologists, of which 85.5% were non-malignant findings.

**Conclusion:**

Validation of radiologists’ findings using computer-aided detection software for automated breast ultrasound has the potential to improve the performance of radiologists. Validation of computer-aided detection might be an efficient tool for double-reading strategies by limiting the amount of discordant cases needed to be double-read.

## Introduction

Population-based breast cancer screening with mammography reduces breast cancer mortality by 31%–48% (1). Nonetheless, in women with dense breasts, sensitivity is as low as 61% (compared to 86% in non-dense breasts). Moreover a 5–6-fold increase in interval cancers is seen in women with extremely dense breasts (2). While modern therapy regimes for breast cancer have improved the life expectancy of breast cancer patients, detecting breast cancer at an early stage is still considered vital for patient survival (3). Women with dense breasts may, therefore, benefit from supplemental imaging modalities to detect mammographically occult cancer.

Breast ultrasound with hand-held ultrasound (HHUS) devices has been shown to help detect mammography-occult early stage invasive breast cancers in women with dense breasts (4–6). However hand-held devices depend highly on the experience of the sonographers and the possibility for comparison of screening exams over time is limited (7). Automated three-dimensional (3D) breast ultrasound (ABUS) devices may overcome the operator dependency of HHUS. The visualization of architectural distortion (the so-called retraction phenomenon (8)) in the coronal plane improves the characterization and detection of breast cancer (9). The acquisition protocols are standardized so that non-sonographers can acquire large 3D whole-breast ultrasound volumes, which can be stored in, and retrieved from, medical imaging archive systems, thus enabling temporal comparison and double-reading strategies. Like supplemental HHUS screening, supplemental ABUS also improves the sensitivity of screening and may likewise lead to an increase of unnecessary recalls because of visualization of benign breast disease that warrants histological evaluation (10–13). The number of ABUS images to read depends on the size of a woman’s breast. A bilateral ABUS examination may consist of 4–10 3D ABUS volumes to ensure coverage; as a consequence, reading ABUS may be a lengthy task and prone to interpretation errors. Double-reading strategies for ABUS may help to prevent interpretation errors (11) but require substantial resources to facilitate.

Computer-aided detection (CAD) systems have shown promising results in breast imaging as an aid for radiologists reading screening mammograms, but in general may lead to an increase in false-positive recalls that need to be dismissed by radiologists (14,15). The conventional implementation of CAD is by marking regions suspicious for cancer in an image and such software has also been developed for ABUS (8,16–18). CAD may help to improve sensitivity, specificity, and/or efficiency of radiologists reading ABUS when implemented as a conventional aid (19,20).

However, there are other ways CAD can be implemented in clinical practice. In this study, we propose using a dedicated ABUS CAD-program to validate findings reported by radiologists during screening for breast cancer in ABUS without primary CAD assistance. Radiologists have been shown to have a relatively high false-positive recall rate when using ABUS. A large proportion of false-positive recalls are caused by benign lesions and ABUS imaging artefacts (21,22). The CAD software used in this study appears to perform well when differentiating malignancies from benign lesions and artefacts. We hypothesized that most recalls for findings that are not recognized as suspicious by the CAD system are based upon artifacts and benign lesions. Therefore, the purpose of this study is to evaluate the effect on the performance of radiologists after using CAD software to validate suspicious findings as pointed out by breast radiologists screening for breast cancer in ABUS.

## Material and Methods

The need for informed consent for using anonymized data in this multi-reader-multi-case (MRMC) observer study was waived by the institutional review board.

We used the data from a previously published MRMC study for the assessment of the added value of a CAD system for validation of findings by radiologists (23). Our previous study (23) reported on the effect of CAD on the accuracy of radiologists using ABUS as a conventional aid. This study focuses on the effect of CAD on radiologists when implemented as a secondary independent interpreter of the radiologists’ findings. As reported elsewhere in detail, cases were extracted from a multi-institutional database containing ABUS examinations from 715 women. In short, the final dataset consisted of 120 unilateral breast examinations (a total of 375 views) with 30 malignant cases, 30 cases containing benign lesions, and 60 normal cases with two years of negative follow-up. All lesions were annotated by a radiologist in training with four years of experience with ABUS, drawing an outline on the lesion edge using in-house built software based on original pathology and radiology reports.

All cases were read twice by eight independent readers with varying levels of experience with ABUS (range = 0–8 years), once without the aid of a CAD system in a standard multiplanar hanging and once with the aid of a commercially developed ABUS CAD software package (QVCAD, Qview Medical Inc., Los Altos, CA, USA). This software is designed to detect suspicious regions in an ABUS volume and mark them. Furthermore, this CAD software package provides an “intelligent” minimum intensity projection (MinIP) of the breast tissue in a 3D ABUS volume that was integrated in the multiplanar hanging protocol. For the current study, only the data from the unassisted readings were used. All readers annotated suspicious lesions by placing a marker in the lesion center and provided a BI-RADS score per case, as well as a level-of-suspiciousness (LOS) score on a linear scale of 0–100 with given anchor points for each BI-RADS assessment category (21, 41, 61, and 81 for BI-RADS category 2, 3, 4, and 5, respectively).

### Validation of findings with CAD software

We used the CAD system, using its default setting of an average of one false-positive CAD region per ABUS volume, for retrospective validation of the reader annotations in the unaided reading session. For this, we recorded the 3D voxel coordinates of each CAD region in the study dataset. At the used threshold, the sensitivity of the CAD system is approximately 82%.

After correlation to CAD findings, reader findings were only considered positive when they corresponded to the location of a CAD region (i.e. positive assessment of findings by both reader and CAD); all other reader findings were regarded as negative (readers marked the finding as positive whereas CAD did not mark the finding). A match was defined as ≤10 mm spherical distance between CAD region and reader marker.

### Evaluation of CAD-rejected findings

To evaluate the type of findings that were rejected with the CAD system (i.e. the negative reader findings after CAD validation), a panel of two experienced readers evaluated in consensus all rejected findings that were reported as ≥BI-RADS 3 by the readers. First, the rejected findings were classified as true negatives (TN) or false negative (FN). FNs were defined as a reader’s marking pointing out a malignant lesion that was rejected by the CAD system. TN findings were findings that were rejected by the system and were not malignant in nature. TNs were subsequently classified in consensus as benign, normal breast tissue, or artefacts.

### Statistical analysis

The area under the alternative free-response operator receiving characteristics (AFROC) curve (AUC) was determined for the unassisted ABUS readings and after CAD validation for each reader individually and for all readers pooled. Only the highest rated lesion per case was included in the analysis. The AFROC analysis included only the LOS scores. False-positive findings in malignant cases were omitted from the analysis to avoid readers and CAD being rewarded while breast cancer was respectively missed or rejected by CAD, which would be the result in a normal case-based ROC analysis and therefore AFROC analysis was chosen.

A full AUC represents all trade-offs between sensitivity and specificity of readers independent of the set of cases and readers. Nevertheless, in screening, a high specificity is required. For that reason, we also analyzed the partial AUC (pAUC) for the false-positive fraction (FPF = 1-specificity) interval of 0.0–0.2 (based on the range in which the specificity of supplemental ultrasound screening has been reported (4,6,12,24–26)). Furthermore, sensitivity for all readers was determined in a simulated sensitivity analysis at a fixed specificity of 90%. PROPROC curve fitting was used to approximate the AUC and pAUC, respectively. MRMC AFROC analysis was performed using the Obuchowski–Rochette Dorfman–Berbaum–Metz MRMC software (v. 2.50) that employs ANOVA and jackknifing (27,28).

Statistical significance was determined if *P* < 0.05.

## Results

### Patient characteristics

Patient characteristics are described in detail in our previous report (23). The average age of women in our dataset was 45.1 years (age range = 26–77 years; SD = 10.4). In the malignant, benign, and normal subcohorts, the average age was 49.8 years (age range = 26–77 years; SD = 12.1), 44.9 years (age range = 30–73 years; SD = 9.1), and 43.0 years (age range = 26–62 years; SD = 9.5), respectively.

The dataset consisted of 84 cases (including 13 malignant and 15 benign cases) that were derived from supplemental screening exams and enriched with 36 exams of symptomatic women. Median cancer size was 14 mm (range = 7–55 mm; SD = 8.8) and median biopsied benign lesion size was 12.4 mm (range = 6–27 mm; SD = 5.1). The subset of cancers consisted of 22 invasive ductal carcinomas, three invasive lobular carcinomas, two invasive intraductal papillary carcinomas, two invasive metaplastic carcinomas, and one invasive apocrine carcinoma. The benign subset consisted of 12 fibroadenomas, two papillomas, three fibrotic lesions, two adenosis, one complex sclerosing lesion, five benign cystic lesions, and five other benign lesions.

### Reader performance

**Fig. 1.**
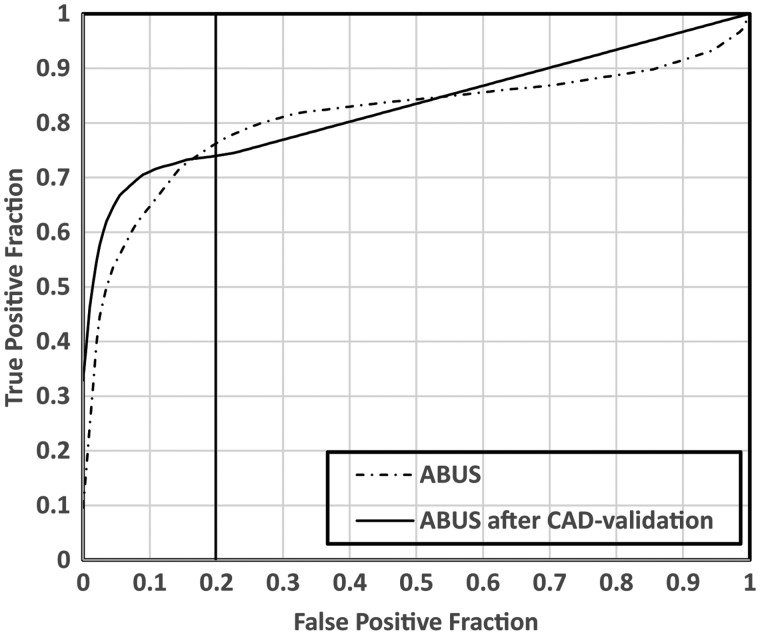
AFROC curves for both standard ABUS reading and results of ABUS reading after CAD validation of human-observed findings. In the higher specificity ranges (false-positive fraction = 1 – specificity) results of reading ABUS with CAD validation outperform standard ABUS reading. The vertical line indicates the specificity of the pAUC estimations are in the range of 80%–100%.

Table 1 summarizes the results of the readers before and after validation of readers’ findings by CAD. The overall difference in AUC was not statistically significant: 0.823 (95% CI = 0.730–0.916) for unaided reading and 0.833 (95% CI = 0.747–0.919) for reading after CAD validation (*P* = 0.743). Validation by CAD improved the partial AUC for the interval within the specificity range of 80%–100%. The pAUC improved significantly from 0.126 (95% CI = 0.098–0.153) to 0.142 (95% CI = 0.115–0.169) (*P* = 0.037) in this specificity interval. Moreover, all readers showed higher pAUC after validation with CAD of which two improved their performance statistically significant. Due to the large reduction of normal findings all readers showed an improvement in sensitivity in a statistical simulation using a fixed specificity of 90% for all radiologists. In fact, pooled sensitivity at 90% specificity was 0.654 (95% CI = 0.512–0.796) for standard ABUS reading and showed improvement to 0.727 (95% CI = 0.598–0.856) after validation by CAD, although the difference was not statistically significant (*P* = 0.061). Fig. 1 shows the pooled AFROC curves for both standard ABUS reading and after validation by CAD. The AFROC curves cross at approximately 83% and 50% specificity likely due to interpolation because of a low number of non-suspicious findings reported after CAD validation.

**Table 1.** Area under the AFROC curves for both standard ABUS reading and results of ABUS after CAD validation for individual readers and pooled over all readers.

Area (FPF interval)	Pooled (95% CI)	Reader 1 (95% CI)	Reader 2 (95% CI)	Reader 3 (95% CI)	Reader 4 (95% CI)	Reader 5 (95% CI)	Reader 6 (95% CI)	Reader 7 (95% CI)	Reader 8 (95% CI)
AUC (0–1)
ABUS	0.823 (0.730–0.916)	0.773 (0.633–0.913)	0.790 (0.656–0.923)	0.732 (0.591–0.872)	0.850 (0.749–0.951)	0.882 (0.784–0.979)	0.874 (0.789–0.958)	0.878 (0.791–0.964)	0.806 (0.693–0.920)
ABUS and CAD- validation	0.833 (0.747– 0.919)	0.833 (0.737–0.930)	0.791 (0.687–0.895)	0.797 (0.694–0.901)	0.853 (0.760–0.947)	0.836 (0.736–0.936)	0.843 (0.748–0.937)	0.861 (0.770–0.952)	0.851 (0.759–0.942)
*ΔSE*	*0.031*	*0.042*	*0.052*	*0.036*	*0.034*	*0.041*	*0.044*	*0.044*	*0.041*
*P*	*0.743*	*0.148*	*0.983*	*0.066*	*0.921*	*0.268*	*0.488*	*0.700*	*0.279*
pAUC (0–0.2)				
ABUS	0.126 (0.098–0.153)	0.104 (0.047–0.161)	0.125 (0.091–0.159)	0.097 (0.055–0.139)	0.138 (0.105–0.172)	0.150 (0.110–0.189)	0.130 (0.098–0.163)	0.138 (0.106–0.170)	0.122 (0.086–0.158)
ABUS and CAD- validation	0.142 (0.115–0.167)	0.139 (0.111–0.167)	0.127 (0.096–0.158)	0.116 (0.064–0.167)	0.148 (0.118–0.178)	0.159 (0.132–0.186)	0.137 (0.102–0.173)	0.163 (0.137–0.189)	0.147 (0.119–0.174)
*ΔSE*	*0.008*	*0.026*	*0.011*	*0.012*	*0.010*	*0.017*	*0.015*	*0.015*	*0.012*
*P*	***0.037***	*0.183*	*0.861*	*0.131*	*0.350*	*0.587*	*0.656*	*0.103*	***0.042***
Sensitivity at 90% specificity				
ABUS	0.654 (0.512–0.796)	0.551 (0.279–0.823)	0.644 (0.467–0.821)	0.513 (0.303–0.724)	0.709 (0.538–0.881)	0.773 (0.594–0.952)	0.687 (0.520–0.854)	0.722 (0.559–0.885)	0.631 (0.448–0.815)
ABUS and CAD validation	0.727 (0.598–0.856)	0.712 (0.578–0.847)	0.653 (0.505–0.802)	0.611 (0.366–0.856)	0.755 (0.614–0.896)	0.806 (0.678–0.935)	0.703 (0.525–0.882)	0.827 (0.704–0.950)	0.749 (0.618–0.879)
*ΔSE*	*0.039*	*0.122*	*0.056*	*0.059*	*0.052*	*0.075*	*0.077*	*0.074*	*0.060*
*P*	*0.061*	*0.190*	*0.870*	*0.101*	*0.384*	*0.655*	*0.833*	*0.157*	*0.052*

AUCs are given for the full AFROC curve and for four intervals in the highest specificity range (FPF = 1 – specificity). The sensitivity at 90% specificity is given for each individual reader and for all readers pooled.

FPF, false-positive fraction (= 1 – specificity); AUC, area under the curve; ΔSE, standard error of the difference between ABUS AUC and CAA AUC.

### Rejected findings after CAD validation

**Fig. 2.**
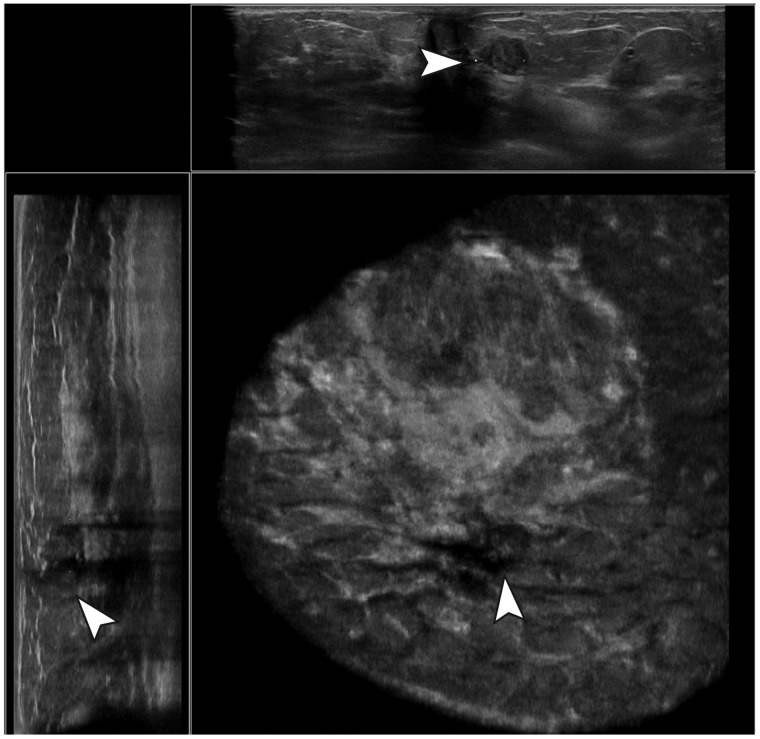
Example of a true-negative case. A hypoechoic, parallel-oriented, well-defined mass with posterior acoustic enhancement and an irregular margin was not marked by the CAD software. Histopathologic evaluation resulted in a fibroadenoma without atypia.

**Fig. 3.**
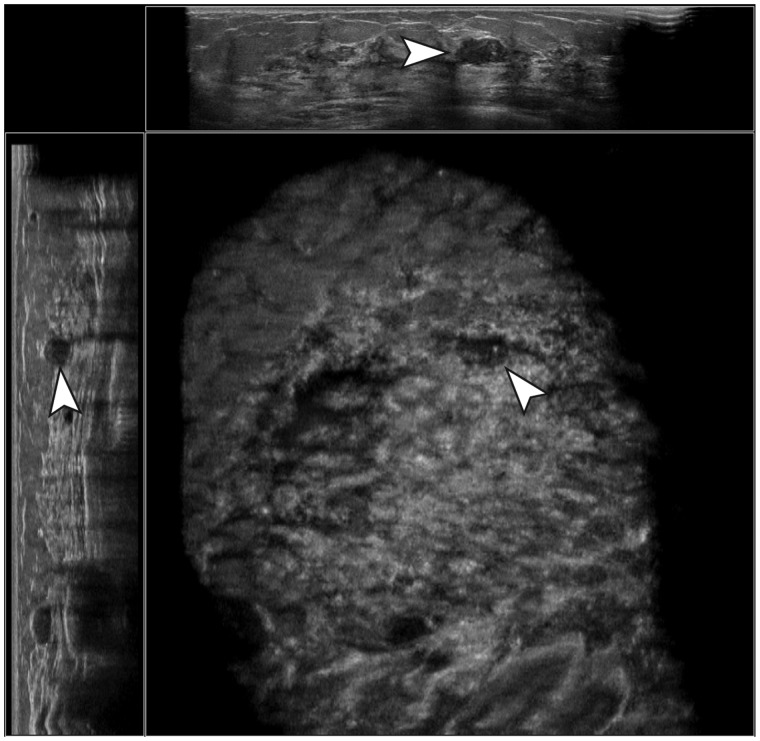
Example of a true-negative case. A hypoechoic, well-defined, parallel-oriented mass with posterior acoustic enhancement, calcifications, and an irregular margin was not marked by the CAD software. Histopathologic evaluation resulted in a fibroadenoma without atypia.

Validation by CAD reduced the number of positive findings, defined as those scored as BI-RADS ≥3 by the readers, with on average 42.6% (range = 31.9%–53.8%) (Figs. 2 and 3). Based on the consensus reading, on average, 49.8% (range = 11.6%–73.3%) of CAD-rejected cases are related to the presence of a benign lesion, 35.7% (range = 13.3%–67.4%) are caused by acoustic shadowing artefacts in normal tissue, and 14.4% (range = 7.7%–20.9%) are malignant lesions missed by the CAD system. The majority (average = 47.5%; range = 37.5%–53.3%) of the CAD-rejected findings were scored by the radiologists as BI-RADS 3, followed by BI-RADS 4 (average = 41.3%; range = 26.7%–58.3%) and BI-RADS 5 (average = 10.4%; range = 4.2%–23.2%) (Table 2).

**Table 2.** CAD-rejected findings (BI-RADS ≥ 3) consensus of final assessment and distribution of BI-RADS scores per radiologist.

Findings BI-RADS ≥3	Reader 1		Reader 2		Reader 3		Reader 4		Reader 5		Reader 6		Reader 7		Reader 8		Mean (%)
CAD-rejected findings* (n (%))	16	(34.8)	20	(43.5)	15	(31.9)	24	(46.1)	18	(35.3)	26	(46.4)	34	(49.3)	43	(53.8)	42.6
Normal tissue/artefacts (n (%))	6/16	(37.5)	5/20	(25.0)	2/15	(13.3)	7/24	(29.2)	5/18	(27.8)	7/26	(26.9)	20/34	(58.8)	29/43	(67.4)	35.7
Benign lesions (n (%))	8/16	(50.0)	11/20	(55.0)	11/15	(73.3)	12/24	(50.0)	11/18	(61.1)	17/26	(65.4)	11/34	(32.3)	9/43	(20.9)	49.8
Malignant lesions (n (%))	2/16	(12.5)	4/20	(20.0)	2/15	(13.3)	5/24	(20.8)	2/18	(11.1)	2/26	(7.7)	3/34	(8.8)	5/43	(11.6)	14.5
CAD-rejected findings (n (%)) BI-RADS score																
3 (n (%))	8/16	(50.0)	10/20	(50.0)	8/15	(53.3)	9/24	(37.5)	9/18	(50.0)	13/26	(50.0)	16/34	(47.1)	18/43	(41.9)	47.5
4 (n (%))	6/16	(37.5)	10/20	(50.0)	5/15	(33.3)	14/24	(58.3)	6/18	(33.3)	11/26	(42.3)	16/34	(47.1)	15/43	(34.9)	42.1
5 (n (%))	2/16	(12.5)	0/20	(0.0)	2/15	(13.3)	1/24	(4.2)	3/18	(16.7)	2/26	(7.7)	2/34	(5.8)	10/43	(23.2)	11.9

*Radiologists read identical sets of ABUS cases; the number of cases reported by radiologists varied.

### Rejected cancers

**Fig. 4.**
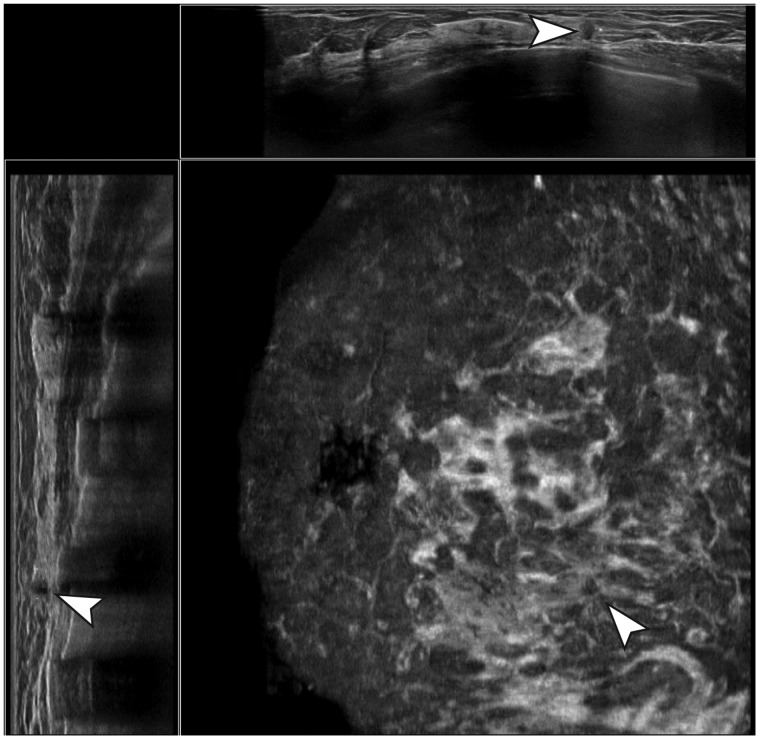
Example of a false-negative case. A small, non-parallel-oriented hypoechoic lesion with irregular margins, no posterior acoustic effects, and a strong retraction pattern was detected and marked correctly by multiple readers, but not by the CAD software and therefore rejected after CAD validation.

Four FN cancers were not marked by CAD at the used threshold of one false-positive/ABUS volume and therefore always led to a rejection when accurately detected by the readers (Fig. 4). Two other FN cancers were correctly identified by CAD, but the extent of the tumor was > 10 mm and as a result the radiologists’ markings were “too far” from the CAD marking in the ABUS volume and therefore did not lead to a positive validation of the radiologists finding. This led to incorrect rejection of malignant findings due to the fact that the spherical distance between reader finding markers and CAD region markers that was used to automatically determine whether CAD marks and reader findings matched was > 10 mm. Finally, two cancers were visible in multiple ABUS volumes but only marked by CAD in one ABUS volume, whereas they were marked by some of the readers in another volume.

## Discussion

Our study shows that implementing CAD software for ABUS as a tool to validate radiologists’ findings has the potential to improve the accuracy of radiologists who use ABUS to detect breast cancer in women with dense breasts, albeit at the cost of slightly lowering sensitivity. Particularly in the highest range of specificities, a significant improvement of the average accuracy 0.126 (95% CI = 0.098–0.153) to 0.142 (95% CI = 0.115–0.169) (*P* = 0.037) is observed. We did not observe an improvement in the overall pooled AUC. Nevertheless, it is important in screening for (breast) cancer to have a method that has high specificity to minimize false-positive screening outcomes that may lead to unnecessary anxiety among the screening participants and negatively impact the cost-effectiveness. A post-hoc analysis of significant (BI-RADS ≥3) but CAD-rejected findings shows that CAD validation may discard 42.6% of findings that were scored BI-RADS ≥3 by the radiologists and 85.5% of the rejected findings were non-malignant and thus were rejected correctly.

Whole breast ultrasound leads to the detection of mammographically occult breast cancer mainly because of visualization of cancers that are masked by fibroglandular tissue (4,10,25,29). Cancers detected by ultrasound tend to be more invasive, node-negative, and smaller in size compared to mammography-detected cancers in screening (30), which may have a positive outcome on patient survival (3). A negative effect of supplemental breast ultrasound is an increase in recall rates, while up to 30% of cancers still could have been detected earlier (31). Choi et al. and Vourtsis et al. showed ABUS in asymptomatic women may outperform hand-held devices in terms of recall rate, but also in terms of cancer detection (9,32). Recently developed CAD software for ABUS may improve screening efficiency, aid radiologists in detecting subtle cancers, and might improve specificity (19,20,23).

Current CAD systems are designed to be implemented as a tool to assist radiologists during evaluation of breast imaging. Such CAD systems may, for example, have a positive effect on the outcome of breast cancer patients that underwent mammographic screening (33). However, CAD systems in mammographic screening have also been criticized because of an increased recall rate induced by CAD (34). Introducing conventional CAD systems into existing breast imaging routines is challenging and depends on several factors, such as the intrinsic accuracy of the CAD system itself and, on a psychological level, the confidence of radiologists in using CAD (35). The latter is likely to be of less importance in alternative ways of CAD implementation such as synthetic lesion enhancement, pre-selection of normal cases for reducing workload, and, according to our study, validation of human observed findings by CAD (36).

Although the CAD system did not detect some of the cancers detected by the readers, and therefore excluded those from further analysis, a fraction of these specific cancers was not rated as very suspicious by the readers and consequently would only have been detected at lower specificity according to the AFROC analyses in this study. In screening, keeping the recall rate at an acceptable level demands a very high specificity. Therefore, we evaluated the average sensitivity per reader at a fixed score of 90% specificity (a statistical simulation based on the LOS scores), which we deem acceptable in screening practice. At a specificity of 90%, the sensitivity for all readers is on average 7% higher, thus suggesting that in practice the use of CAD might allow a higher cancer detection in screening, based on better selection of recalled cases. In an ideal situation, radiologists would recall all women with breast abnormalities with a certain degree of suspiciousness. But population-based screening should be both accurate and affordable; therefore, some population-based screening programs have restrictions on the number of recalls in order to achieve a positive cost-effectiveness ratio. Our results might imply that using CAD validation may allow radiologist to recall more women initially (by lowering their specificity) and potentially might improve their sensitivity.

**Fig. 5.**
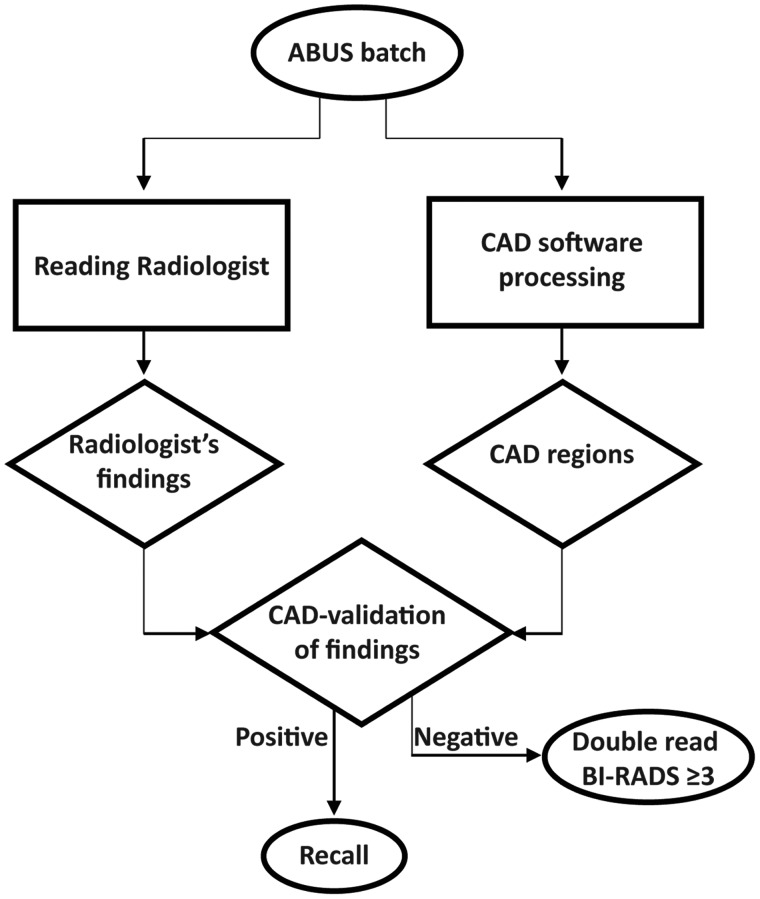
Flow diagram of an example of an ABUS screening workflow that incorporates CAD validation of human-observed findings and reduces the workload of double-reading discordant cases.

In an alternative and clinically more acceptable scenario, CAD validation might be used in a double-reading strategy. This prevents unwanted rejection of malignant cases, while still largely reducing the workload for the second reader as only discordant cases need to be reviewed to optimize screening performance. Fig. 5 shows a schematic workflow of a theoretical double-reading strategy of ABUS that includes CAD as a validation tool. The effect of combinations of CAD and double-reading of discordant cases in ABUS requires further investigation.

To our knowledge, mammographic screening programs that offer supplemental ultrasound to women with dense breasts have not implemented CAD systems. Wilczek et al. used consensus arbitration of discordant cases in double-reading to reduce false-positive recall rates to an acceptable level (11). According to our results, CAD validation of radiologists’ findings may positively affect the false-positive recall rate and thus achieve similar screening sensitivity at higher screening specificity.

Our study has limitations. The prevalence of both benign and malignant breast disease was artificially enhanced to increase power of this study and does not resemble normal screening practice where disease prevalence is lower. ABUS and mammography are usually complementary; however, we did not show mammography to the readers which may have affected the results either positively or negatively. Furthermore, readers in our study were unexperienced with batch reading large quantities of ABUS exams which may have affected individual recall strategies.

In conclusion, in this paper we presented results of CAD validation of radiologists’ findings in ABUS using commercially developed dedicated CAD software. CAD has the potential to help radiologists avoid unnecessary recalls by validating radiologists’ reports in screening. CAD validation may be integrated into double-reading strategies and consequently might reduce the resources needed for double-reading of ABUS by confirming cases that were found suspicious and leaving only non-CAD suspicious cases for double-reading. Whether validation of findings with CAD actually improves the screening performance and reduces the costs for double-reading needs further prospective investigation.
